# Concentration-Dependent Rheological Properties of Atelocollagen Are Associated with Fibroblast Mechanotransduction and Collagen Remodeling in Aged Skin

**DOI:** 10.3390/jfb17090474

**Published:** 2026-09-17

**Authors:** Seyeon Oh, Gwahn Woo Cheon, Jae Ik Lee, Hyoung Moon Kim, Min Seung Kim, Soo Jeong Heo, Kuk Hui Son, Kyunghee Byun

**Affiliations:** 1 Functional Cellular Networks Laboratory, Lee Gil Ya Cancer and Diabetes Institute, Gachon University, Incheon 21999, Republic of Korea; 2 LIBON Inc., Incheon 22006, Republic of Korea; 3 Department of Anatomy & Cell Biology, College of Medicine, Gachon University, Incheon 21936, Republic of Korea; 4 Maylin Clinic, Ilsan, Goyang 10391, Republic of Korea; 5 Department of Thoracic and Cardiovascular Surgery, International Healthcare Center, Seoul National University Bundang Hospital, Seongnam 13620, Republic of Korea; 6 Maylin Apgujeong Clinic, Seoul 06024, Republic of Korea; 7 Inee Clinic, Seoul 06030, Republic of Korea; 8 Magma Clinic, Seoul 06014, Republic of Korea; 9 Department of Thoracic and Cardiovascular Surgery, Gachon University Gil Medical Center, College of Medicine, Gachon University, Incheon 21565, Republic of Korea; 10 Department of Health Sciences and Technology, Gachon Advanced Institute for Health & Sciences and Technology (GAIHST), Gachon University, Incheon 21999, Republic of Korea

**Keywords:** atelocollagen, oscillatory rheology, mechanotransduction, integrin β1, collagen remodeling

## Abstract

Injectable collagen biomaterials are used to modify the dermal extracellular matrix in aged skin; however, whether concentration-dependent rheological differences are associated with distinct fibroblast responses remain unclear. We compared 3% and 6% atelocollagen (AtCOL) and examined three mechanobiological programs: (i) integrin (ITG) α5β1–ERK–cyclin D1 signaling related to proliferation; (ii) ITGβ1–FAK–YAP signaling related to matrix synthesis; and (iii) ITGα11β1/Tensin-1–positive fibrillar adhesion related to collagen assembly. Under the tested oscillatory shear conditions, 6% AtCOL displayed higher storage modulus (G′), loss modulus (G″), and complex viscosity, together with a lower tan δ, than 3% AtCOL. In aged mouse skin, the 6% formulation was associated with greater increases in ITGα5, pERK1/2, cyclin D1, PCNA, pFAK, nuclear YAP, COL1A1, COL3A1, ITGα11β1/Tensin-1 co-expression signal, collagen type I/III ratio, collagen type I fiber bundle width, mature collagen content, dermal collagen density, and an instrument-derived skin elasticity index, while MMP1, MMP2, and MMP9 were reduced. In an H_2_O_2_-induced fibroblast senescence model, ITGβ1 knockdown attenuated AtCOL-associated proliferative, matrix-synthetic, and fibrillar-adhesion responses. Complementary ITGβ1 overexpression produced directionally concordant increases in ITGA11 expression, relative fibroblast proliferation, COL1A1, and COL3A1 and decreases in MMP1, MMP2, and MMP9. Collectively, these findings are consistent with a concentration-dependent, ITGβ1-centered mechanotransduction model linking fibroblast proliferation, matrix synthesis, and collagen assembly within a single AtCOL system.

## 1. Introduction

Skin aging is not merely a quantitative loss of collagen; it reflects progressive failure of the dermal extracellular matrix (ECM) microenvironment. In youthful skin, dermal fibroblasts are anchored to an organized, collagen-rich matrix that provides tensile support, preserves cell spreading, and sustains normal matrix homeostasis [1,2,3,4,5]. Through intrinsic aging and photoaging, collagen fibrils become fragmented, fibroblast–ECM interactions weaken, fibroblasts lose their elongated morphology and mechanical force, collagen production declines, and levels of matrix-degrading enzymes (e.g., matrix metalloproteinases [MMPs]) increase, establishing a self-perpetuating cycle of dermal deterioration [1,2,3,4,5]. At the tissue level, skin is a viscoelastic structure; age-related changes in elastic recoil, stiffness, and other time-dependent mechanical properties have been documented, indicating that mechanical degradation is a central component of skin aging rather than a secondary consequence [1,2,3,4,5].

Accordingly, fibroblasts respond to biochemical ligands within the ECM and to its physical properties. Of particular importance is viscoelasticity: the time-dependent combination of elastic energy storage and viscous energy dissipation during deformation [6,7,8,9]. In native tissues and engineered matrices, viscoelasticity determines how long cell-generated forces are maintained, how focal adhesions mature, and how efficiently cells remodel their surroundings [6,7,8].

Recent studies have shown that, even when the elastic modulus is similar, changes in viscoelastic behavior can substantially alter fibroblast spreading, adhesion strength, traction generation, migration, proliferation, and Yes-associated protein (YAP) activity [8]. Matrix viscoelasticity is now recognized as an active regulator of cell behavior, rather than merely a passive material descriptor [6,7,8].

These physical cues are interpreted through integrin (ITG)-based mechanotransduction pathways. The ITGα5β1 axis is particularly relevant to proliferation-associated matrix sensing because ITGα5β1-mediated adhesion can sustain extracellular signal-regulated kinase (ERK) signaling and induce cyclin D1 expression [9]. ITGβ1 more broadly acts as a mechanosignaling hub in fibroblasts; in mechanically stimulated dermal fibroblasts, it regulates focal adhesion kinase (FAK) phosphorylation, and FAK–Src signaling promotes YAP nuclear localization. Given that YAP/TAZ are mechanoactivated regulators of fibroblast proliferation, activation, and matrix synthesis, the ITGβ1–FAK–YAP pathway provides a plausible mechanism linking ECM mechanics to collagen production [10,11,12,13]. In parallel, ITGα11β1 is induced in a mechanosensitive manner; recent work has shown that it localizes to Tensin-rich fibrillar adhesions and functions as a collagen assembly receptor at the cell surface [14]. Collectively, these data suggest that ECM physical properties regulate fibroblast behavior through at least three coordinated programs: proliferative entry, force-transducing matrix synthesis, and fibrillar adhesion-mediated collagen assembly.

These concepts are highly relevant to injectable fillers. Prior human studies of cross-linked hyaluronic acid (HA) have shown that filler injection can mechanically stretch dermal fibroblasts, increase procollagen expression, and stimulate de novo collagen deposition in aged or photodamaged skin [15]. A randomized study similarly linked cross-linked HA to dermal mechanical stress and a measurable biochemical response; recent human research demonstrated fibroblast elongation and thick collagen bundle accumulation after HA injection [16]. Additional studies indicate that filler products with different compositions or rheologic profiles do not produce identical biological effects: commercial HA fillers can induce varying magnitudes of collagen and elastin expression; higher-viscosity, higher-elasticity HA–polynucleotide complexes can stimulate greater fibroblast migration and collagen synthesis than HA alone; and calcium hydroxylapatite (CaHA) can induce a remodeling pattern distinct from that of HA [17]. Most prior studies have focused on HA-based products, compared different commercial formulations, or emphasized clinical performance rather than directly interrogating concentration-driven viscoelastic differences within a collagen filler system.

Given this background, we hypothesized that an increased atelocollagen (AtCOL) concentration would alter filler rheology and modulate fibroblast collagen-producing capacity. Specifically, we postulated that the higher-concentration formulation would enhance three convergent mechanobiological programs: (i) ITGα5β1–ERK–cyclin D1 signaling linked to fibroblast proliferation; (ii) ITGβ1–FAK–YAP signaling linked to matrix synthesis; and (iii) ITGα11β1/Tensin-1–positive fibrillar adhesion linked to collagen assembly. To test this hypothesis, we compared 3% and 6% AtCOL formulations in a skin-aging model after subcutaneous administration, then examined rheologic behavior together with mechanotransduction markers, collagen synthesis, collagen organization, and matrix remodeling.

## 2. Materials and Methods

### 2.1. Preparation of AtCOL Formulations

Type I AtCOL-based formulations (Laetigen) were manufactured by Pharvis Korea Co., Ltd. (Seoul, Republic of Korea) using porcine-derived AtCOL supplied by Dmed, LLC (Seongnam, Republic of Korea). The AtCOL component was prepared as previously described [18,19,20,21]. Two formulations containing 3% and 6% (*w*/*v*) AtCOL were used as the test materials in the present study.

### 2.2. Rheological Analysis

The rheological properties of AtCOL samples were evaluated using a rotational rheometer (ARES-G2, TA Instruments, New Castle, DE, USA). The analysis was performed by the Korea Polymer Testing & Research Institute (KOPTRI, Seoul, Republic of Korea). AtCOL samples at concentrations of 3% and 6% were subjected to rheological measurements. All measurements were conducted at 25 ℃ using a 40 mm parallel-plate geometry. Oscillatory shear measurements were performed at a constant strain of 100% over a frequency range of 0.1–100 Hz. For quantitative comparison between the 3% and 6% AtCOL samples, values obtained at a frequency of 1 Hz were used. The storage modulus (G′), loss modulus (G″), and complex viscosity (η*) were recorded and analyzed using the manufacturer-provided software (version 5.1; TA Instruments, New Castle, DE, USA). These measurements characterize the formulations under the applied oscillatory shear conditions. Stress-relaxation, creep/recovery, and long-term deformation tests were not performed.

### 2.3. In Vivo Experiments

Male C57BL/6 mice (10–11.5 months old) were purchased from Orient Bio Inc. (Seongnam, Republic of Korea) and maintained under standard laboratory conditions (22 ± 2 ℃, 50–60% humidity, 12 h light/dark cycle) with ad libitum access to food and water. All animals were naturally aged; to minimize age-related variability, all groups were sampled at 12 months of age. A separate group of young male C57BL/6 mice (8 weeks old; Orient Bio Inc., Seongnam, Republic of Korea) was used as a reference group to confirm age-associated changes in the naturally aged model. The young mice were not included in the AtCOL treatment experiment and were used only for validation of the aging phenotype. For the AtCOL injection experiment, the experimental unit was a single animal, and mice were randomly assigned to seven groups (*n* = 5 per group; total *n* = 35) [22]. No animals or data points were excluded from the analysis. This study was approved by the Institutional Animal Care and Use Committee of Gachon University (approval number: LCDI-2026-0005 and date of approval 28 January 2026), and all procedures were conducted in accordance with the ARRIVE guidelines. All animal experiments were performed in an AAALAC-accredited facility in compliance with relevant institutional and international guidelines for animal care and use.

AtCOL or saline was administered subcutaneously to the dorsal skin at a total volume of 700 µL per animal over an approximately 4 cm^2^; injection area, divided among seven injection sites (100 µL per site) [18]. The same injection scheme was used for all groups. Mice were anesthetized by isoflurane inhalation; dorsal hair was removed using a razor and depilatory cream before injection. All injections were performed using a sterile syringe, and all tissue samples were collected from the injection sites. Investigators performing outcome assessments were blinded to group allocation where possible.

The experimental groups were defined based on AtCOL concentration (3% and 6%) and observation period (2, 4, and 8 weeks). A saline-treated aged group served as a control. The groups were defined as follows: Group 1, saline-injected aged mice with tissue collection at 8 weeks; Group 2, aged mice injected with 3% AtCOL and sampled at 2 weeks; Group 3, aged mice injected with 3% AtCOL and sampled at 4 weeks; Group 4, aged mice injected with 3% AtCOL and sampled at 8 weeks; Group 5, aged mice injected with 6% AtCOL and sampled at 2 weeks; Group 6, aged mice injected with 6% AtCOL and sampled at 4 weeks; and Group 7, aged mice injected with 6% AtCOL and sampled at 8 weeks.

### 2.4. Skin Elasticity Index Assessment

An instrument-derived skin elasticity index was assessed using a Skin Analyzer (API 100; Aram Huvis, Seoul, Republic of Korea). Measurements were performed using a non-contact optical imaging method to capture high-resolution images of the dorsal skin surface at the injection sites. Elasticity index values were calculated using the manufacturer-provided analysis software (Solutionist v1.7.10, Aram Huvis, Seoul, Republic of Korea) under standardized conditions.

To minimize variability, all measurements were conducted at the same anatomical location for each animal immediately prior to tissue sampling. Each mouse was measured five times, and the average elasticity index was used for subsequent analysis.

### 2.5. In Vitro Experiments

#### 2.5.1. Cell Culture and Senescence Induction

Human dermal fibroblasts (CEFO™ Human Dermal Fibroblast Cells, CEFO-HF; CEFO Co., Ltd., Seoul, Republic of Korea) were used between passages 5 and 8. Cells were cultured in CEFOgro™ Human MSC Growth Medium (CEFO Co., Ltd., Seoul, Republic of Korea) and maintained at 37 ℃ in a humidified incubator with 5% CO_2_.

For senescence induction, human dermal fibroblasts were seeded at 1 × 10^6^ cells in 100 mm dishes and cultured for 48 h, then treated with H_2_O_2_ (350 µM; Sigma-Aldrich, St. Louis, MO, USA) for 1.5 h. After treatment, cells were washed with Dulbecco’s phosphate-buffered saline (DPBS; Thermo Fisher Scientific, Waltham, MA, USA) to remove residual H_2_O_2_, and the medium was replaced with fresh growth medium. Cells were further incubated for 72 h to establish an H_2_O_2_-induced senescence model [23]. In parallel, control cells were treated with DPBS under the same conditions. Senescence induction was confirmed by quantitative real-time PCR analysis of p16 and p21 expressions and by senescence-associated β-galactosidase (SA-β-gal) assay.

#### 2.5.2. AtCOL Treatment Conditions

To evaluate cytotoxicity, senescent human dermal fibroblasts were treated with AtCOL at concentrations of 0.1, 0.5, 1, 5, 10, and 15 mg/mL for 48 h. Cells were seeded at 5 × 10^4^ cells per well in 96-well plates and allowed to attach overnight prior to treatment.

AtCOL working solutions were prepared by diluting the stock solution in DPBS and subsequently mixing with serum-free medium to achieve the final treatment concentrations. Cell viability was assessed using a cell viability assay kit (TransDetect^®^ Cell Counting Kit; TransGen Biotech Co., Ltd., Beijing, China) in accordance with the manufacturer’s instructions, and the non-cytotoxic concentration range was determined.

Based on the cytotoxicity results, additional experiments were conducted using lower concentrations of AtCOL (0, 50, 100, 200, and 400 µg/mL) for 48 h to further evaluate suitable treatment conditions within the non-cytotoxic range.

For subsequent in vitro experiments, the 6% AtCOL formulation used in the in vivo study was diluted in culture medium to a final AtCOL concentration of 400 µg/mL. This concentration was selected based on its acceptable cytocompatibility and the greater efficacy of the 6% formulation observed in the in vivo experiments. Control groups received equivalent volumes of DPBS under the same conditions.

#### 2.5.3. ITGβ1 Knockdown and AtCOL Treatment

To investigate the role of ITGβ1 in the effects of AtCOL, senescent HDFs were transfected with ITGβ1-targeting shRNA plasmids (Santa Cruz Biotechnology, Inc., Dallas, TX, USA) using Lipofectamine™ 3000 Transfection Reagent (Thermo Fisher Scientific, Waltham, MA, USA) according to the manufacturer’s instructions.

After 24 h of transfection, cells were treated with AtCOL or PBS. Cells were divided into four experimental groups as follows:

(1) Control senescent cells treated with PBS (Control/PBS);

(2) Control senescent cells treated with AtCOL (Control/AtCOL);

(3) ITGβ1-knockdown senescent cells treated with PBS (ITGβ1-KD/PBS);

(4) ITGβ1-knockdown senescent cells treated with AtCOL (ITGβ1-KD/AtCOL).

Following treatment, cells were incubated for 48 h and then harvested for downstream analyses.

#### 2.5.4. ITGβ1 Overexpression and AtCOL Treatment

The effect of increased ITGβ1 expression on the response of senescent HDFs to AtCOL was assessed using an ITGβ1 overexpression plasmid encoding human ITGB1 with a C-terminal Myc-DDK tag (OriGene Technologies, Rockville, MD, USA). The corresponding empty pCMV6-Entry vector (OriGene Technologies) was used as the vector control. Plasmids were introduced into senescent HDFs using Lipofectamine™ 3000 Transfection Reagent (Thermo Fisher Scientific, Waltham, MA, USA) in accordance with the manufacturer’s instructions.

After 24 h post-transfection, the cells were treated with either AtCOL or PBS vehicle. Four groups were analyzed:

(1) empty vector-transfected cells treated with PBS (EV/PBS);

(2) empty vector-transfected cells treated with AtCOL (EV/AtCOL);

(3) ITGβ1-overexpressing cells treated with PBS (ITGβ1-OE/PBS);

(4) ITGβ1-overexpressing cells treated with AtCOL (ITGβ1-OE/AtCOL).

After a further 48 h of incubation, cells were harvested for subsequent analyses.

### 2.6. Quantitative Analysis of Senescence Marker Gene Expression

Total RNA was isolated from cells and tissues using RNAiso reagent (Takara Bio Inc., Shiga, Japan) according to the manufacturer’s instructions. cDNA was synthesized from 1 µg of total RNA using a cDNA synthesis kit (a cDNA synthesis kit; Takara Bio Inc., Shiga, Japan) according to the manufacturer’s instructions. Quantitative PCR was subsequently carried out using SYBR Green chemistry (Takara Bio Inc., Shiga, Japan) with gene-specific primers targeting p16 and p21, together with Actb as the internal reference gene. Relative transcript levels were determined using the 2^-ΔΔCt^ method, with expression values first normalized to ACTB and subsequently expressed relative to those of the Non-SnCs or Young groups. The primer sequences used in this study are listed in Table S1.

### 2.7. SA-β-Gal Assay

Senescence was assessed using a SA-β-gal kit (Cells Signaling Technology, Danvers, MA, USA) according to the manufacturer’s instructions. SA-β-gal positive cells were identified by blue cytoplasmic staining. Bright-field images were acquired under identical imaging conditions, and the SA-β-gal signal was quantified using ImageJ software (version 1.54g; National Institutes of Health, NIH, Bethesda, MD, USA). The quantified values were normalized to those of the Non-SnCs group or Young group and expressed as relative SA-β-gal signal.

### 2.8. Cell Proliferation Assay

Cell proliferation was assessed using the cell viability assay kit (TransDetect^®^ Cell Counting Kit; TransGen Biotech Co., Ltd., Beijing, China). HDFs were plated in 96-well plates at a density of 1 x 10^5^ cells per well and cultured overnight to allow cell attachment. The following day, cells were exposed to the designated treatments according to the experimental schedule for each model and cultured for the indicated duration. At each assessment time point, 10 µL of cell counting kit reagent was added to each well containing 100 µL of culture medium. The plates were incubated for 4 h at 37 ℃ in a humidified atmosphere containing 5% CO_2_. Absorbance was measured at 450 nm, and proliferation was expressed relative to the corresponding control group for each experimental condition.

### 2.9. Protein Extraction and Immunoblotting

Protein samples were obtained from cells and tissues using a lysis buffer containing protease and phosphatase inhibitors (ATTO Corporation, Tokyo, Japan). Total protein concentrations were quantified using a bicinchoninic acid assay (Thermo Fisher Scientific).

For immunoblot analysis, proteins (30 µg per sample) were resolved on sodium dodecyl sulfate–polyacrylamide gels and subsequently transferred to polyvinylidene difluoride membranes (Merck Millipore, Sigma-Aldrich, St. Louis, MO, USA). EasySee^®^ Western Marker and EasySee^®^ II Western Marker (TransGen Biotech Co., Ltd., Beijing, China), covering ranges of 25–90 kDa and 30–150 kDa, respectively, were used as standards to verify the apparent molecular weights of the detected protein bands. Membranes were incubated in a blocking solution that comprised 5% skim milk in Tris-buffered saline with 0.1% Tween-20 for 1 h at room temperature. After membranes had been blocked, they were incubated with specific primary antibodies (Table S2) at 4 ℃ overnight. After a washing step, membranes were incubated with horseradish peroxidase-conjugated secondary antibodies (Vector Laboratories, Newark, CA, USA) for 1 h at room temperature. Immunoreactive signals were detected using chemiluminescent substrates (Cytiva, Marlborough, MA, USA) and captured using an imaging system. Band intensities were analyzed using ImageJ software (1.54g; NIH, Bethesda, MD, USA), and target protein levels were normalized to β-actin. Relative expression levels were calculated by comparing each group with its respective control.

### 2.10. Enzyme-Linked Immunosorbent Assay (ELISA)

Protein samples were prepared in carbonate–bicarbonate coating buffer (pH 9.6; Sigma-Aldrich, St. Louis, MO, USA) and applied to 96-well plates (SPL Life Sciences, Pocheon, Republic of Korea), then incubated at 4 ℃ overnight to allow protein binding. Plates were then washed with PBS containing 0.1% Tween-20 and blocked with 5% skim milk (LPS Solution, Daejeon, Republic of Korea) for 1 h at room temperature.

After the blocking step, plates were incubated overnight at 4 ℃ with primary antibodies targeting COL1A1, COL3A1, ITGα5/β1, and ITGα11/β1 (see Table S2 for details). After plates had been washed with PBS containing 0.1% Tween-20, horseradish peroxidase-conjugated secondary antibodies (1:1000; Vector Laboratories, Newark, CA, USA) were added to wells and incubated for 1 h at room temperature.

Enzymatic color development was performed using 3,3′,5,5′-tetramethylbenzidine substrate (Sigma-Aldrich, St. Louis, MO, USA), and the reaction was terminated by adding 1 M sulfuric acid (Merck Millipore, Sigma-Aldrich, St. Louis, MO, USA). Optical density was measured at 450 nm using a microplate reader (Multiskan SkyHigh; Thermo Fisher Scientific, Waltham, MA, USA).

Relative protein levels were calculated based on absorbance values and expressed as fold changes normalized to the corresponding control group.

### 2.11. Preparation of Paraffin-Embedded Skin Sections

Excised skin tissues were immediately fixed in 4% paraformaldehyde (Sigma-Aldrich, St. Louis, MO, USA) at 4 ℃ for 72 h. After fixation, samples were placed in tissue cassettes and rinsed with distilled water to remove residual fixative.

The tissues were subsequently processed through a graded series of ethanol solutions for dehydration, then cleared in xylene (Duksan Pure Chemicals Co., Ltd., Ansan, Republic of Korea). Next, dehydrated samples were infiltrated with molten paraffin (Leica, Wetzlar, Germany) and embedded to generate paraffin blocks.

Paraffin-embedded tissues were sectioned at a thickness of 7 µm using a microtome (Thermo Fisher Scientific, Waltham, MA, USA). Sections were mounted on coated glass slides (Muto Pure Chemicals Co., Ltd., Tokyo, Japan) and dried overnight at 60 ℃ prior to histological staining.

### 2.12. Immunohistochemistry

Paraffin-embedded skin sections were deparaffinized with xylene and rehydrated through a graded ethanol series. Tissue permeability was enhanced by incubation with 0.5% Triton X-100 (Sigma-Aldrich, St. Louis, MO, USA) for 5 min, followed by washing with PBS.

To minimize nonspecific binding, sections were blocked with normal serum (Vector Laboratories, Newark, CA, USA) for 1 h at room temperature. Slides were then incubated overnight at 4 ℃ with PCNA and YAP antibodies (Table S2) diluted in blocking buffer. After thorough washing, sections were treated with biotinylated secondary antibodies (Vector Laboratories, Newark, CA, USA) for 1 h at room temperature.

Signal detection was performed using an avidin–biotin complex system (Vector Laboratories, Newark, CA, USA) according to the manufacturer’s instructions, followed by visualization with a chromogenic substrate. Nuclei were counterstained with hematoxylin (KPNT, Cheongju, Republic of Korea). Sections were then dehydrated through a graded ethanol series, cleared in xylene, and mounted using DPX mounting medium (Sigma-Aldrich, St. Louis, MO, USA).

Slide images were acquired using a digital slide scanner (SLIDEVIEW VS200, Olympus, Tokyo, Japan), and representative regions were selected for analysis. The extent of 3,3′-diaminobenzidine-positive staining was quantified using ImageJ software (1.54g; NIH, Bethesda, MD, USA). Specifically, the proportion of brown-stained nuclei within the dermal region was measured and expressed as fold changes relative to the saline control group.

### 2.13. Immunofluorescence Analysis

Paraffin-embedded skin sections (7 µm thickness) were first deparaffinized in xylene and subsequently rehydrated through a descending ethanol gradient (100% to 70%), then rinsed in distilled water. Antigen retrieval was performed by heat-induced epitope retrieval in citrate buffer (pH 6.0; Sigma-Aldrich, St. Louis, MO, USA) using a microwave until boiling, after which the sections were allowed to cool on ice.

Sections were washed with PBS and, where required for intracellular or nuclear localization analysis, permeabilized with 0.5% Triton X-100 in PBS for 5 min at room temperature. Nonspecific binding was blocked by incubating the sections with a serum-containing blocking solution for 1 h at room temperature.

Primary antibodies (listed in Table S2) were applied to the sections and incubated overnight at 4 ℃. After sections had been washed with PBS, they were incubated with Alexa Fluor 488- or 594-conjugated secondary antibodies (Invitrogen, Thermo Fisher Scientific, Waltham, MA, USA) at a dilution of 1:500 for 1 h at room temperature. Nuclear staining was performed using 4′,6-diamidino-2-phenylindole (DAPI, 1 µg/mL; Sigma-Aldrich, St. Louis, MO, USA) for 20 s.

After a final washing step, sections were mounted with fluorescence mounting medium (Vectashield; Vector Laboratories, Newark, CA, USA). Fluorescent images were acquired using a slide fluorescence imaging system (SLIDEVIEW VS200, Olympus, Tokyo, Japan) under identical acquisition settings for all experimental groups. Fluorescence intensity, positive cell counts, and collagen fiber bundle width were quantified using OlyVIA software (version 4.2; Olympus, Tokyo, Japan) and ImageJ software (version 1.54g; NIH, Bethesda, MD, USA). For nuclear localization analysis, the proportion of positively stained nuclei was determined within defined regions of interest. For collagen fiber analysis, the apparent width of clearly discernible collagen type I-immunoreactive fiber bundles width was measured in micrometers, perpendicular to the longitudinal axis of each fiber bundle [24], within predefined dermal regions of interest.

### 2.14. Immunocytochemical Analysis

Cells were cultured and subjected to senescence induction as described above. After treatment under the indicated experimental conditions, cells were fixed with 4% paraformaldehyde in PBS for 15 min at room temperature and rinsed with PBS. To facilitate intracellular staining, cells were permeabilized with 0.5% Triton X-100 in PBS for 10 min and subsequently incubated with a serum-based blocking solution (Vector Laboratories, Newark, CA, USA) for 1 h at room temperature to reduce nonspecific binding. Cells were then incubated overnight at 4 ℃ with primary antibodies (Table S2) diluted in blocking buffer. After cells had been washed with PBS, Alexa Fluor-conjugated secondary antibodies (Invitrogen, Thermo Fisher Scientific, Waltham, MA, USA) were applied for 1 h at room temperature in the dark. Nuclear counterstaining was performed using DAPI (Sigma-Aldrich, St. Louis, MO, USA). After a final washing step, cells were coverslipped using a mounting medium, and fluorescent images were obtained using a slide fluorescence imaging system (SLIDEVIEW VS200, Olympus, Tokyo, Japan). Quantitative analysis of fluorescence signals was conducted using OlyVIA software (version 4.2; Olympus, Tokyo, Japan). Fluorescence intensity and the number of positively stained cells were measured; for nuclear localization analysis, the proportion of marker-positive nuclei was calculated within defined regions of interest.

### 2.15. Herovici Staining

Differential staining of collagen fibers was performed using a commercial Herovici staining kit (ScyTek Laboratories, Logan, UT, USA), in accordance with the manufacturer’s protocol. Briefly, paraffin-embedded sections were stained with Weigert’s iron hematoxylin for nuclear visualization, then incubated with Herovici staining solution to distinguish collagen subtypes.

After staining, sections were dehydrated through a graded ethanol series, cleared in xylene, and mounted with a permanent mounting medium. Slide images were acquired using a digital slide scanner, and representative fields were selected for analysis.

For quantitative assessment, newly synthesized collagen fibers (blue) and mature collagen fibers (red) were analyzed separately [25,26]. Color deconvolution was applied using ImageJ software (1.54g; NIH, Bethesda, MD, USA) to isolate each staining component. The relative density of each collagen subtype was measured within the dermal region and expressed as fold changes normalized to the saline control group.

### 2.16. Masson’s Trichrome Staining

Collagen deposition in skin tissues was evaluated using a modified Masson’s trichrome staining kit (ScyTek Laboratories, Logan, UT, USA) following the manufacturer’s instructions. After staining, sections were dehydrated through a graded ethanol series, cleared in xylene, and coverslipped using a permanent mounting medium.

Stained sections were digitized using a slide imaging system, and representative regions were selected for analysis. Collagen fibers were identified based on blue staining, and quantitative analysis was performed using ImageJ software (1.54g; NIH, Bethesda, MD, USA).

For quantification, collagen-positive areas within the dermal region were isolated using color deconvolution. The proportion of collagen-stained areas relative to the total dermal area was calculated and expressed as fold changes compared with the young/saline control group.

### 2.17. Statistical Analysis

All quantitative data are expressed as mean ± standard deviation. Given the small sample size in each group, nonparametric statistical methods were utilized for all analyses. Group comparisons were performed using the Kruskal–Wallis test, followed by post hoc pairwise comparisons using the Mann–Whitney U test with Bonferroni correction for multiple comparisons. Statistical analyses were conducted via SPSS software (version 26; IBM, Armonk, NY, USA). Information regarding sample size, statistical significance, and the number of biological replicates is provided in the corresponding figure legends. Bonferroni-adjusted *p*-values < 0.05 were considered statistically significant.

## 3. Results

### 3.1. Rheological Characterization Shows That 6% AtCOL Has Higher G′, G″, and Complex Viscosity Under the Tested Oscillatory Shear Conditions

**Figure 1:**
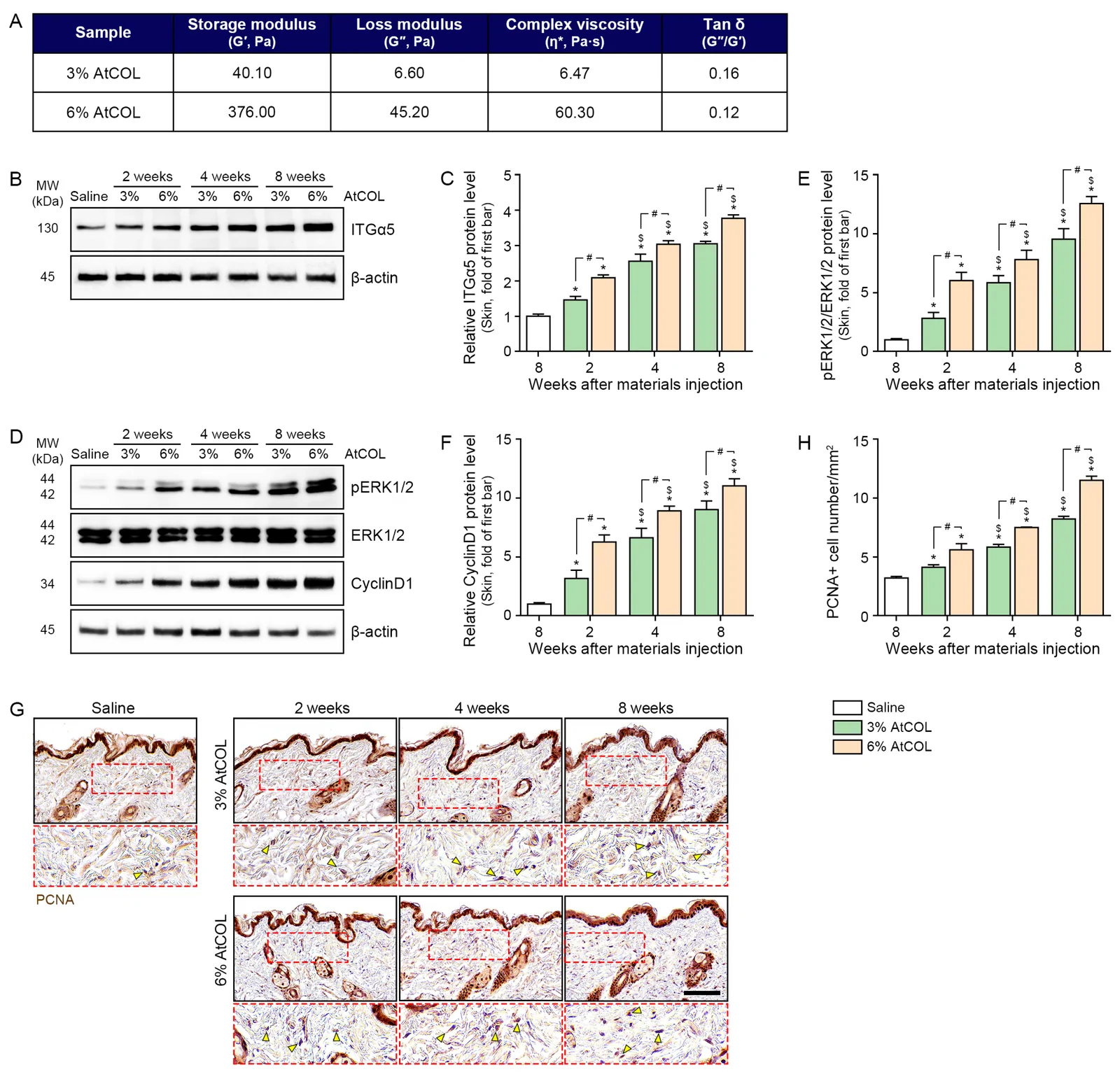
AtCOL promotes fibroblast proliferation in aged skin through activation of the ITGα5–ERK signaling pathway. (**A**) Rheological properties of AtCOL at 3% and 6% concentrations. Storage modulus (G′), loss modulus (G″), complex viscosity (η*), and loss tangent (tan δ; G″/G′) were measured to characterize oscillatory shear behavior. The value shown represent measurements obtained at 1 Hz. A higher G′ indicates greater elastic energy storage, whereas a higher G″ indicates greater energy dissipation under oscillatory deformation. Tan δ values < 1 indicate an elastic-dominant response under the tested conditions. These parameters do not assess stress relaxation or creep. (**B**) Representative Western blot images showing ITGα5 expression in aged skin at 2, 4, and 8 weeks after subcutaneous injection of 3% and 6% AtCOL. β-actin was used as a loading control. (**C**) Quantification of ITGα5 protein expression shown in (B), normalized to β-actin and expressed as fold changes relative to the saline-treated group. (**D**) Representative Western blot images showing expression levels of pERK1/2, ERK1/2, and cyclin D1 in aged skin after AtCOL treatment. (**E,F**) Densitometric analysis of pERK1/2/ERK1/2 (**E**) and cyclin D1 (**F**) expression shown in (D), normalized to β-actin and expressed as fold changes relative to the control group. (**G**) Immunohistochemical staining for PCNA in skin sections from saline- and AtCOL-treated groups at the indicated time points. Brown staining indicates PCNA-positive cells (yellow arrow). Scale bar = 100 µm. (**H**) Quantification of PCNA-positive cells in the dermal region shown in (**G**). The number of PCNA-positive nuclei was counted within defined dermal areas and expressed as fold changes relative to the saline-treated group. Data are expressed as mean ± standard deviation (*n* = 5 biologically independent sample per group). Overall group differences were assessed using the Kruskal–Wallis test, followed by predefined pairwise comparisons using the Mann–Whitney U test with Bonferroni correction for multiple comparisons. * indicates a significant difference compared with the saline group, # indicates a significant difference between the 3 and 6% AtCOL groups at the corresponding time point, and $ indicates a significant difference from the preceding time point within the same AtCOL concentration (Bonferroni-adjusted *p* < 0.05). AtCOL, atelocollagen; ITG, integrin; PCNA, proliferating cell nuclear antigen.

Rheological analysis showed that the 6% formulation exhibited higher G′, G″, and η* than the 3% formulation (376 vs. 40.1 Pa, 45.2 vs. 6.60 Pa, and 60.3 vs. 6.47 Pa·s, respectively); both formulations had tan δ < 1. The 6% formulation also showed a lower tan δ than the 3% formulation (0.12 vs. 0.16), indicating a more elastic-dominant response under the applied oscillatory conditions (Figure 1A). These measurements establish differences in oscillatory shear behavior but do not characterize stress relaxation or creep.

### 3.2. The 6% AtCOL Formulation Is Associated with Stronger ITGα5–ERK–Cyclin D1 Signaling and Dermal Proliferative Responses in Aged Skin

The aging phenotype was further supported by increased p16 and p21 mRNA expression in aged skin compared with young (8-week-old) skin (Figure S1). We next evaluated whether the higher-concentration formulation was associated with stronger activation of ITGα5-related proliferative signaling in the aged dermis. Western blot analysis demonstrated that ITGα5 expression increased after AtCOL treatment in a time-dependent manner; the increase was consistently greater in the 6% group than in the 3% group at 2, 4, and 8 weeks (Figure 1B,C). A similar pattern was observed for pERK1/2/ERK1/2 and cyclin D1, both of which were progressively elevated after AtCOL injection and were more strongly induced by 6% AtCOL than by 3% AtCOL at each matched time point (Figure 1D–F).

To determine whether these signaling changes were accompanied by a proliferative response, proliferating cell nuclear antigen (PCNA) immunostaining was performed in the dermis. PCNA-positive cell number increased after AtCOL treatment relative to the saline control; this increase was consistently greater in the 6% group than in the 3% group, with the strongest response observed at 8 weeks (Figure 1G,H). Collectively, these findings show that the 6% formulation, which exhibited higher oscillatory moduli and complex viscosity under the tested conditions, was associated with a stronger ITGα5–ERK–cyclin D1 proliferative response in aged skin.

### 3.3. The 6% AtCOL Formulation Is Associated with Greater FAK–YAP Signaling and Collagen Synthesis in Aged Skin

**Figure 2:**
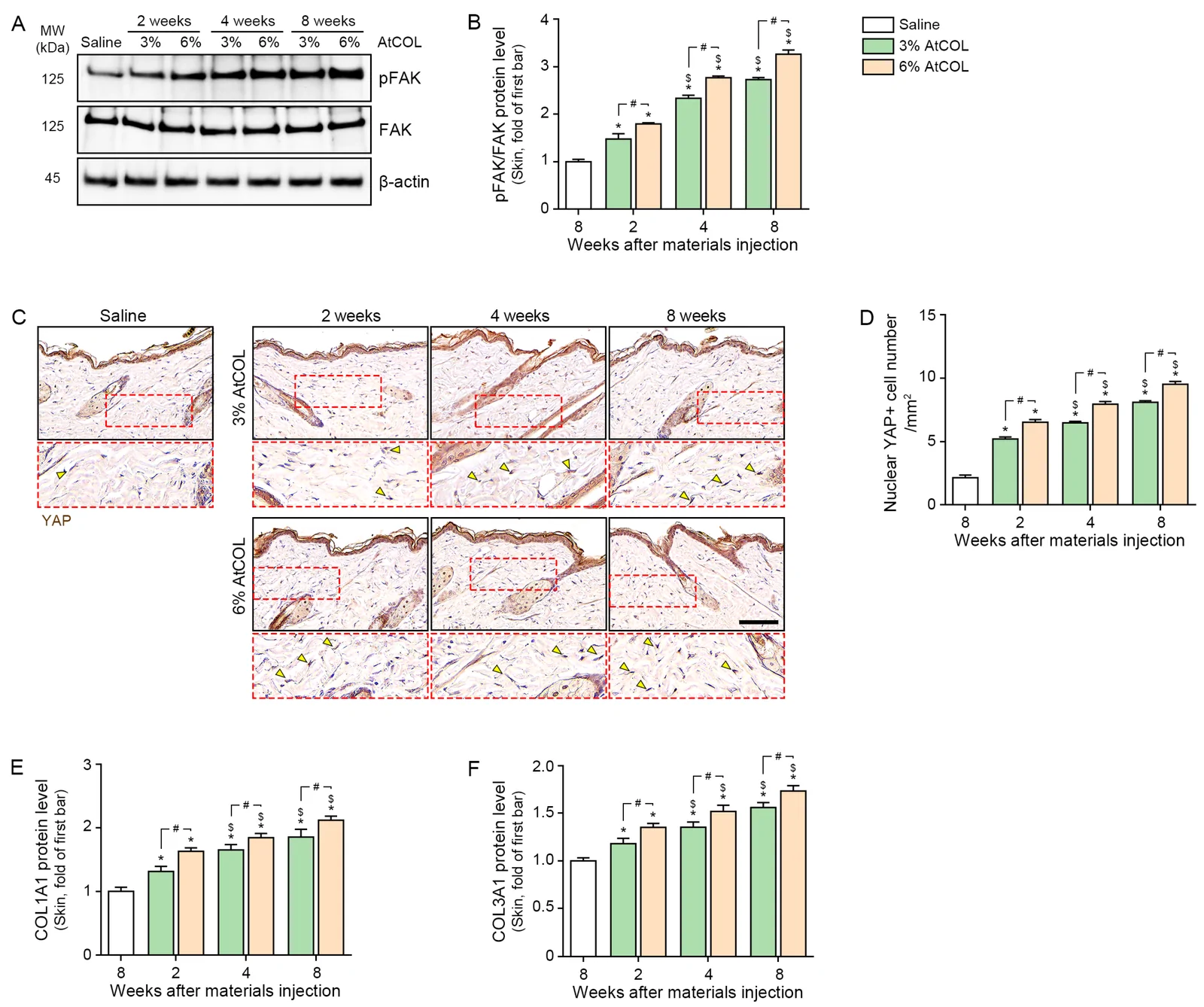
AtCOL enhances collagen synthesis in aged skin through activation of the FAK–YAP signaling pathway. (**A**) Representative Western blot images showing pFAK and total FAK expression in aged skin at 2, 4, and 8 weeks after subcutaneous injection of 3% and 6% AtCOL. β-actin was used as a loading control. (**B**) Quantification of pFAK/FAK levels shown in (A). Protein expression levels were normalized to β-actin and expressed as fold changes relative to the saline-treated group. (**C**) Immunohistochemical staining of YAP in skin sections from saline- and AtCOL-treated groups at the indicated time points. Nuclear localization of YAP is indicated by brown staining (yellow arrow). Representative high-magnification images of the dermal region are shown in the lower panels. Scale bar = 100 µm. (**D**) Quantification of nuclear YAP-positive cells in the dermis shown in (*C*). The number of nuclear YAP-positive cells was counted within defined dermal regions and expressed as fold changes relative to the saline-treated group. (**E**) Quantitative analysis of COL1A1 protein levels measured by ELISA in skin tissues after AtCOL treatment. (**F**) Quantitative analysis of COL3A1 protein levels measured by ELISA in skin tissues after AtCOL treatment. Data are presented as the mean ± standard deviation (*n* = 5 per group). Statistical significance was determined using the Kruskal–Wallis test followed by Mann–Whitney U test with Bonferroni correction. *, *p* < 0.05 vs. saline; #, *p* < 0.05, 3% vs. 6% AtCOL at the same time point; $, *p* < 0.05 vs. the preceding time point within the same AtCOL concentration. All *p*-values shown are Bonferroni-adjusted. AtCOL, atelocollagen; COL1A1, collagen type 1 alpha 1 chain; COL3A1, collagen type 3 alpha 1 chain; ELISA, enzyme-linked immunosorbent assay pFAK, phosphorylated FAK.

In aged skin, 6% AtCOL induced higher pFAK/FAK expression than 3% AtCOL and was accompanied by a greater number of nuclear YAP-positive dermal cells (Figure 2A–D). ELISA analyses showed increased collagen type 1 alpha 1 chain (COL1A1) and collagen type 3 alpha 1 chain (COL3A1) protein levels after AtCOL treatment, again with greater increases in the 6% group (Figure 2E,F). These data demonstrate concentration-dependent differences in FAK activation, YAP nuclear localization, and collagen production in the aged dermis.

### 3.4. The 6% AtCOL Formulation Shows Greater ITGα11β1/Tensin-1-Associated Matrix Remodeling in Aged Skin

**Figure 3:**
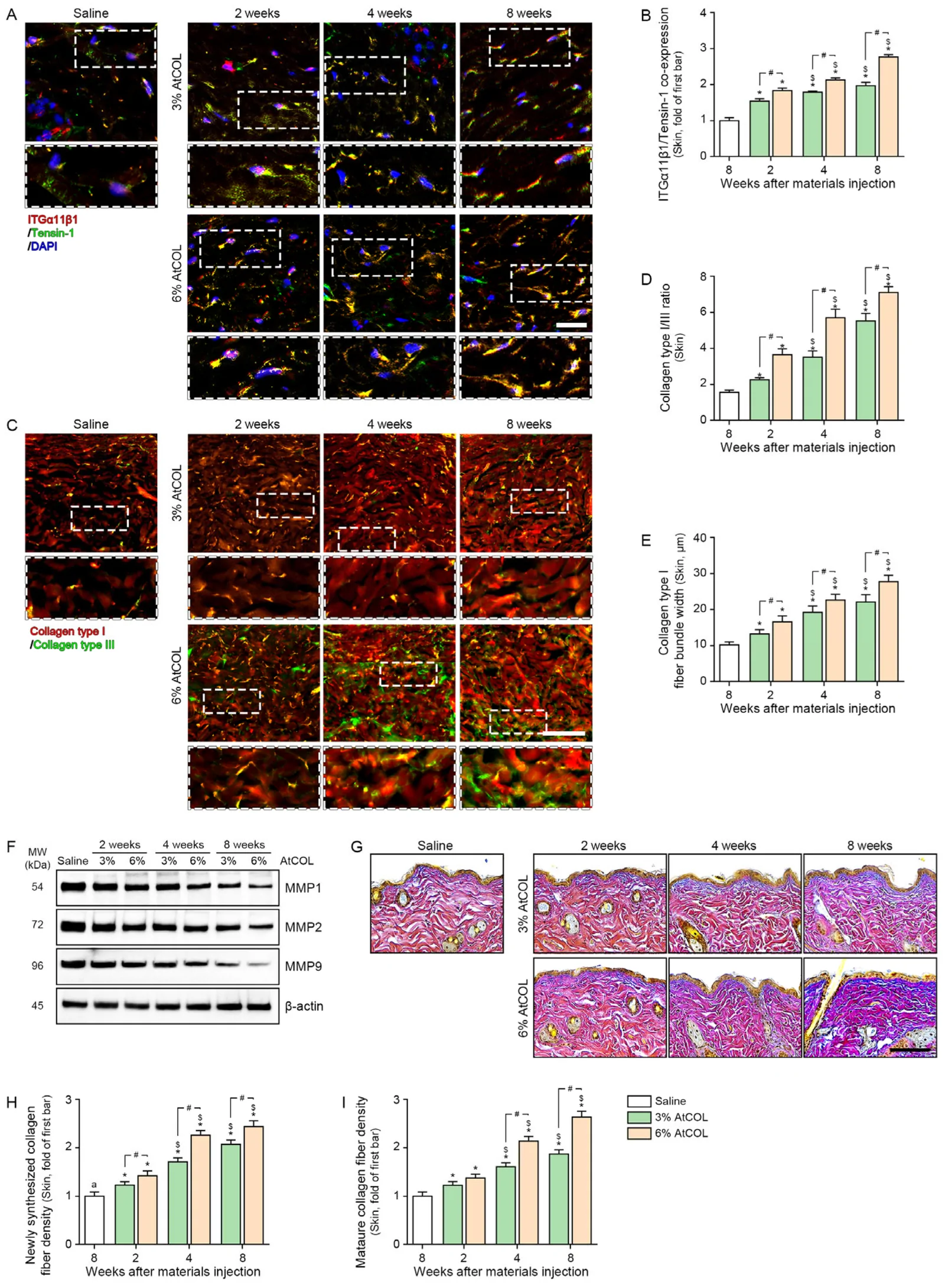
AtCOL regulates extracellular matrix remodeling in aged skin via ITGα11β1-mediated signaling. (**A**) Representative immunofluorescence images showing co-expression of ITGα11β1 (red) and Tensin-1 (green) in skin tissues at 2, 4, and 8 weeks after subcutaneous injection of 3% and 6% AtCOL. Nuclei were counterstained with DAPI (blue). Dashed boxes indicate regions shown at higher magnification in the corresponding enlarged images. Scale bar = 50 µm. (**B**) Quantification of ITGα11β1/Tensin-1 fluorescence co-expression intensity shown in (**A**). Relative expression levels were calculated and expressed as fold changes compared with the saline-treated group. (**C**) Representative immunofluorescence images showing co-staining of collagen type I (red) and collagen type III (green) in skin tissues from each group. Dashed boxes indicate regions shown at higher magnification in the corresponding enlarged images. Scale bar = 50 µm. (**D**) Quantitative analysis of the collagen type I/III ratio based on images shown in (**C**). (**E**) Quantification of collagen type I fiber thickness derived from (**C**). Collagen fiber thickness was measured within the dermal region. (**F**) Representative Western blot images showing expression levels of MMP1, MMP2, and MMP9 after AtCOL treatment. Quantitative analysis is provided in Figure S2. (**G**) Representative Herovici staining images illustrating newly synthesized collagen fibers (blue) and mature collagen fibers (red) in skin sections. Scale bar = 100 µm. (**H**) Quantification of newly synthesized collagen fibers based on Herovici staining shown in (**G**). (**I**) Quantification of mature collagen fibers based on Herovici staining shown in (**G**). Data are expressed as the mean ± standard deviation (*n* = 5 per group). Statistical significance was assessed using the Kruskal–Wallis test followed by Bonferroni-corrected Mann–Whitney U test. *, *p* < 0.05 vs. saline; #, *p* < 0.05, 3% vs. 6% AtCOL at the same time point; $, *p* < 0.05 vs. the preceding time point within the same AtCOL concentration. AtCOL, atelocollagen; DAPI, 4′,6-diamidino-2-phenylindole; ITG, integrin; MMP, matrix metalloproteinases.

We next examined whether AtCOL also enhanced ECM-remodeling pathways linked to collagen assembly. Immunofluorescence staining revealed that ITGα11β1/Tensin-1 co-expression increased after AtCOL treatment and was consistently greater in the 6% group than in the 3% group across the examined time points; the highest merged intensity was observed at 8 weeks (Figure 3A,B).

We then assessed whether these adhesion-related changes were accompanied by alterations in collagen organization. Co-staining for collagen types I and III showed that AtCOL treatment progressively raised the collagen type I/type III ratio, with a more pronounced increase in the 6% group than in the 3% group. In parallel, quantitative image analysis demonstrated that collagen type I fiber bundle width increased after AtCOL treatment, again with a stronger effect in the 6% group, particularly at later time points (Figure 3C–E). These data suggest that the higher-concentration formulation promotes not only collagen accumulation but also a shift toward a more type I collagen-dominant and structurally mature matrix.

In contrast, MMP1, MMP2, and MMP9 levels decreased after AtCOL exposure, with the greatest suppression in the 6% condition (Figure 3F and S2). These data show that the 6% formulation was associated with a greater collagen-remodeling response, including increased assembly-associated adhesion signals and reduced matrix degrading enzymes.

Herovici staining further supported this interpretation. The number of newly synthesized and mature collagen fibers both increased after AtCOL treatment; the magnitude of increase was greater in the 6% group than in the 3% group (Figure 3G–I). Taken together, these results indicate that higher-concentration AtCOL promotes a more active collagen-remodeling response characterized by increased ITGα11β1/Tensin-1-associated matrix remodeling, enhanced collagen maturation, and progressive accumulation of organized collagen fibers in aged skin.

### 3.5. ITGβ1 Knockdown Attenuates AtCOL-Associated Proliferation and Collagen Synthesis in an H_2_O_2_-Induced Fibroblast Senescence Model

**Figure 4:**
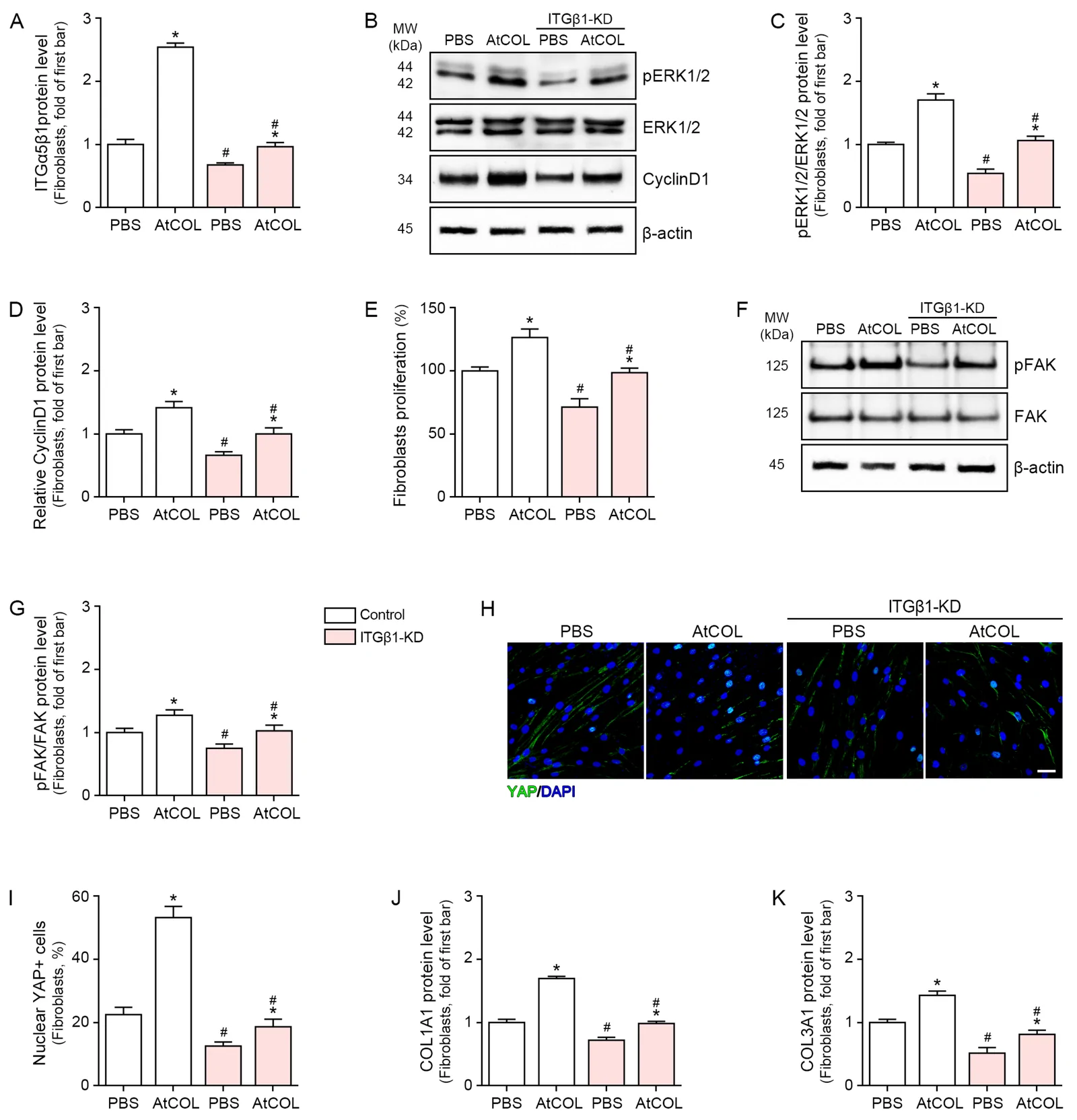
ITGβ1 inhibition attenuates AtCOL-associated fibroblast proliferation and collagen synthesis. H_2_O_2_-treated HDFs in the senescence model were exposed to AtCOL under control or ITGβ1-knockdown conditions. Cells were transfected with ITGβ1-KD prior to treatment and subsequently exposed to AtCOL for 48 h. (**A**) Quantitative analysis of ITGα5β1 expression measured by ELISA after AtCOL treatment. (**B**) Representative Western blot images showing expression patterns of pERK1/2, ERK1/2, and cyclin D1 in control and ITGβ1-KD-treated cells. (**C,D**) Densitometric quantification of pERK1/2/ERK1/2 (**C**) and cyclin D1 (**D**) protein levels shown in (B), normalized to β-actin and expressed as fold changes relative to the PBS-treated control group. (**E**) Cell proliferation assay showing effects of AtCOL treatment with or without ITGβ1 knockdown. (**F**) Representative Western blot images of pFAK and total FAK expression in each group. (**G**) Quantification of pFAK/FAK levels shown in (**F**), normalized to β-actin and expressed as fold changes relative to the control group. (**H**) Immunocytochemical staining of YAP (green) showing nuclear localization in control and ITGβ1-KD-treated cells. Nuclei were counterstained with DAPI (blue). Scale bar = 50 µm. (**I**) Quantification of nuclear YAP-positive cells shown in (**H**). The number of YAP-positive nuclei was determined and expressed as fold changes relative to the PBS-treated control group. (**J**) Quantitative analysis of COL1A1 protein levels measured by ELISA. (**K**) Quantitative analysis of COL3A1 protein levels measured by ELISA. Values are expressed as mean ± standard deviation (*n* = 5 biologically independent sample per group). Statistical significance was assessed using the Kruskal–Wallis test followed by Mann–Whitney U test with Bonferroni correction for multiple comparisons. * indicates a significant difference between PBS and AtCOL within the same conditions, and # indicates a significant difference between control and ITGβ1-KD groups within the same treatment group (Bonferroni-adjusted *p* < 0.05). AtCOL, atelocollagen; COL1A1, collagen type 1 alpha 1 chain; COL3A1, collagen type 3 alpha 1 chain; DAPI, 4′,6-diamidino-2-phenylindole; ELISA, enzyme-linked immunosorbent assay; HDFs, human dermal fibroblasts; ITG, integrin; KD, knockdown; PBS, phosphate-buffered saline; pFAK, phosphorylated FAK.

H_2_O_2_-treated HDFs showed significantly increased P16 and P21 mRNA expression and elevated SA-β-gal level relative to PBS-treated controls (Figure S3), supporting establishment of a senescence-like phenotype. To assess ITGβ1 dependence, H_2_O_2_-treated HDFs were exposed to 6% AtCOL after ITGβ1 knockdown. AtCOL increased ITGα5β1 expression, pERK1/2/ERK1/2, cyclin D1, fibroblast proliferation, pFAK/FAK, nuclear YAP, COL1A1, and COL3A1 in control cells; each response was attenuated by ITGβ1 knockdown (Figure 4). These findings indicate that ITGβ1 contributes to the proliferative and matrix-synthetic response to AtCOL.

### 3.6. ITGβ1 Knockdown Attenuates ITGα11β1/Tensin-1-Positive Fibrillar Adhesion and MMP Regulation in the H_2_O_2_-Induced Senescence Model

**Figure 5:**
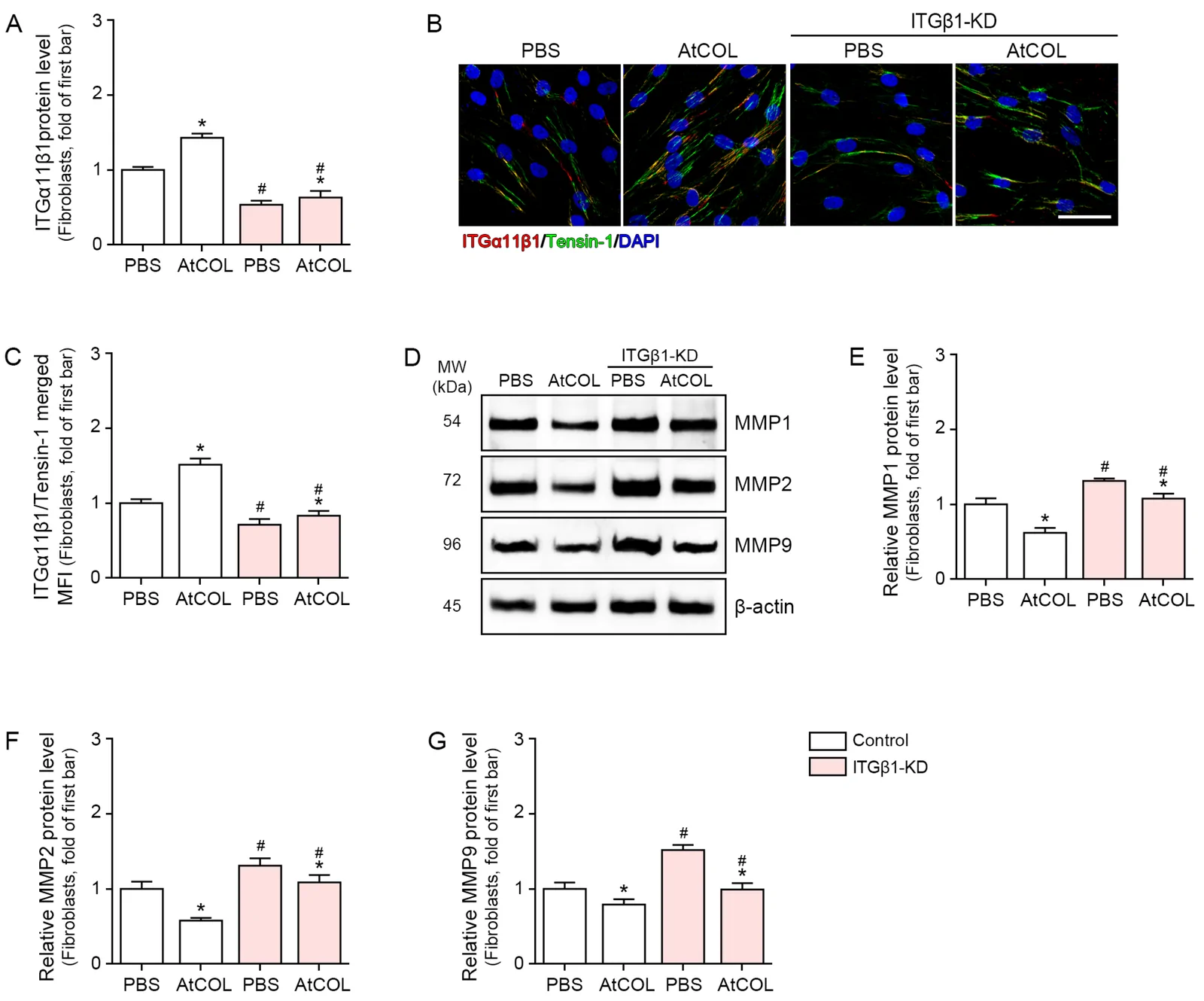
ITGβ1 inhibition modulates fibrillar adhesion and matrix remodeling in the H_2_O_2_-induced fibroblast senescence model. H_2_O_2_-treated HDFs were exposed to AtCOL in the presence or absence of ITGβ1 knockdown. Cells were transfected with ITGβ1-KD and subsequently exposed to 6% AtCOL for 48 h. (**A**) Quantitative analysis of ITGα11β1 expression measured by ELISA after AtCOL treatment under control and ITGβ1-silenced conditions. (**B**) Representative immunocytochemical images showing co-expression of ITGα11β1 (red) and Tensin-1 (green) in fibroblasts. Nuclei were counterstained with DAPI (blue). Scale bar = 50 µm. (**C**) Quantification of ITGα11β1/Tensin-1 co-expression shown in (**B**). Merged fluorescence intensity was measured and expressed as fold changes relative to the PBS-treated control group. (**D**) Representative Western blot images showing expression patterns of MMP1, MMP2, and MMP9 in control and ITGβ1-KD cells. (**E**–**G**) Densitometric analysis of MMP1 (**E**), MMP2 (**F**), and MMP9 (**G**) expression levels shown in (**D**), normalized to β-actin and expressed as fold changes relative to the control group. Data are expressed as mean ± standard deviation (*n* = 5 biologically independent sample per group). Statistical comparisons were performed using the Kruskal–Wallis test followed by Bonferroni-corrected Mann–Whitney U tests. *, *p* < 0.05, PBS vs. AtCOL within the same condition; #, *p* < 0.05, control vs. ITGβ1-KD within the same treatment group. AtCOL, atelocollagen; DAPI, 4′,6-diamidino-2-phenylindole; ELISA, enzyme-linked immunosorbent assay; HDFs, human dermal fibroblasts; ITG, integrin; KD, knockdown; MMP, matrix metalloproteinases; PBS, phosphate-buffered saline.

In H_2_O_2_-treated HDFs, ITGα11β1/Tensin-1 merged intensity increased with 6% AtCOL but decreased after ITGβ1 knockdown. In the same setting, AtCOL-associated suppression of MMP1, MMP2, and MMP9 was lost or attenuated when ITGβ1 was silenced (Figure 5). These results indicate that β1-containing integrin signaling contributes to fibrillar-adhesion-associated remodeling and MMP regulation in this model.

### 3.7. ITGβ1 Overexpression Provides Complementary Gain-of-Function Support for AtCOL Associated Collagen Remodeling

In H_2_O_2_-treated HDFs, ITGβ1 overexpression produced a downstream pattern that was directionally concordant with the knockdown experiment. Compared with PBS controls, ITGβ1-overexpressing cells showed higher ITGA11 transcript abundance and relative fibroblast proliferation, higher COL1A1 and COL3A1 expression, and lower MMP1, MMP2, and MMP9 expression. AtCOL treatment further increased the positive remodeling markers and maintained or enhanced MMP suppression in the ITGβ1-overexpression condition (Figure S4).

### 3.8. AtCOL Increases Dermal Collagen Density and the Skin Elasticity Index in Aged Skin

**Figure 6:**
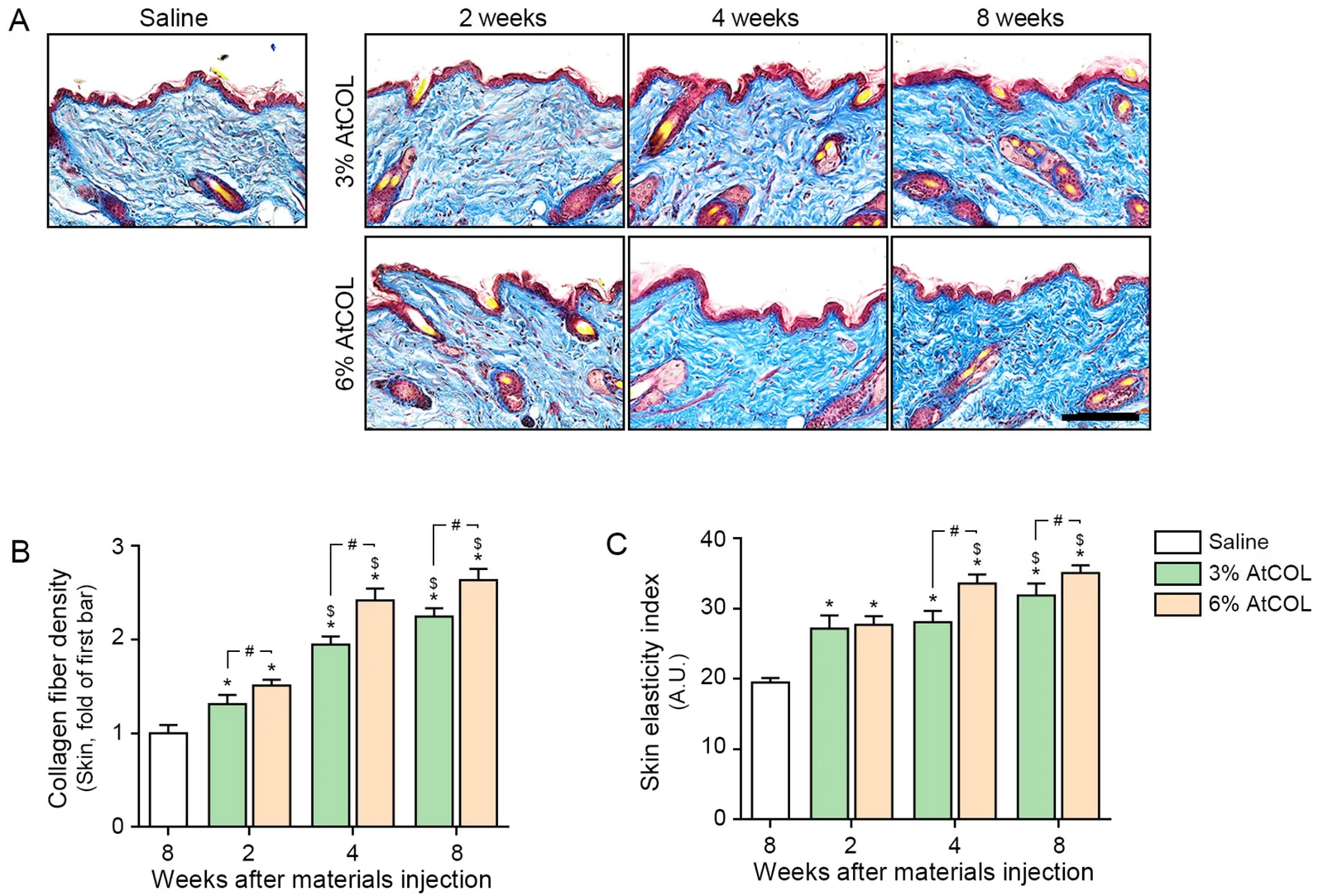
AtCOL increases dermal collagen density and the skin elasticity index in aged skin. (**A**) Representative Masson’s trichrome staining images of skin sections from saline- and AtCOL-treated groups (3% and 6%) at 2, 4, and 8 weeks. Collagen fibers are stained blue, indicating dermal collagen deposition. Scale bar = 100 µm. (**B**) Quantification of collagen fiber content based on Masson’s trichrome staining shown in (**A**). Collagen-positive areas within the dermis were measured and expressed as fold changes relative to the saline-treated group. (**C**) Instrument-derived skin elasticity index after AtCOL treatment. Values were assessed using a skin analyzer and expressed as relative changes compared with the control group. Values are expressed as mean ± standard deviation (*n* = 5 biologically independent sample per group). Statistically significance was determined using the Kruskal–Wallis test followed by Bonferroni-adjusted Mann–Whitney U test. *, *p* < 0.05 vs. saline; #, *p* < 0.05, 3% vs. 6% AtCOL at the same time point; $, *p* < 0.05 vs. the preceding time point within the same AtCOL concentration. AtCOL, atelocollagen.

Masson’s trichrome staining showed greater collagen fiber density in AtCOL-treated dermis than in saline controls, with the strongest response in the 6% group (Figure 6A,B). The instrument-derived skin elasticity index followed the same pattern (Figure 6C).

## 4. Discussion

The present study compared two concentrations of the same AtCOL system and identified a coherent concentration-dependent pattern across oscillatory rheology, signaling, and matrix outcomes. In our subcutaneous injection model, the 6% formulation showed higher G′, G″, and complex viscosity and a lower tan δ than 3% AtCOL and was associated with greater ITGα5–ERK–cyclin D1 signaling, PCNA positivity, pFAK and nuclear YAP, COL1A1/COL3A1, ITGα11β1/Tensin-1 co-expression signal, collagen fiber organization and maturation, and lower MMP1/2/9. ITGβ1 knockdown attenuated AtCOL-associated proliferative, matrix-synthetic, and fibrillar-adhesion responses, whereas ITGβ1 overexpression produced directionally concordant changes in ITGA11, proliferation, collagen transcripts, and MMP expression; together, these data support an ITGβ1-centered model of collagen remodeling but do not establish that a single rheological parameter caused the biological differences.

From a biomaterials perspective, filler performance should not be reduced to chemical composition alone. Fillers are viscoelastic materials; under oscillatory testing, G′ reflects elastic energy storage, G″ reflects energy dissipation, tan δ (G″/G′) describes their relative contribution, and complex viscosity reflects frequency-dependent resistance to oscillatory flow [27,28,29,30]. These parameters describe material behavior under the applied oscillatory loading and may influence resistance to cell-generated traction, integrin clustering, focal adhesion signaling, and YAP localization [6,9,10,11,12,13,14]. Therefore, filler rheology may contribute to the local mechanical microenvironment encountered by resident fibroblasts, but the present measurements do not comprehensively characterize in vivo viscoelastic behavior. Accordingly, the higher G′, G″, and complex viscosity of 6% AtCOL provide a plausible physical context for the stronger remodeling phenotype; however, because stress-relaxation and creep/recovery were not measured, in vivo stress dissipation, long-term deformation, and dynamic load retention cannot be inferred.

Mechanistically, these findings are consistent with ITGα5β1-mediated ERK persistence and cyclin D1 induction as a link to proliferative expansion [9]. They are also consistent with ITGβ1-associated FAK–YAP signaling in force-responsive matrix synthesis [12,31] and with ITGα11β1 localization to Tensin-1-positive fibrillar adhesions during collagen assembly [14]. The attenuation of these responses after ITGβ1 knockdown and the directionally concordant downstream pattern after ITGβ1 overexpression provide complementary loss- and gain-of-function support for coordinated involvement of β1-containing integrins; nevertheless, FAK, YAP, and α11β1 were not individually perturbed, the overexpression experiment did not include the complete FAK–YAP and Tensin-1 panel, and the serial order of the three modules remains unresolved.

Taken together, prior mechanobiology data make it plausible that a collagen filler with a different oscillatory rheological profile could alter force transmission through ITGβ1 and thereby influence ERK, YAP, and fibrillar-adhesion-associated responses. The broader filler literature supports the principle that filler-associated mechanical support can stimulate dermal collagen production [15,16,17]. Human studies of cross-linked HA have reported fibroblast stretching, procollagen induction, and de novo collagen deposition after injection [15,16,17]. These studies provide contextual support for mechanically transmitted fibroblast activation but do not isolate rheology as the causal variable.

Furthermore, prior work indicates that different filler formulations do not generate identical fibroblast responses. In an in vitro comparison of commercial HA fillers, products induced different patterns of collagen and elastin expression [32]. Similarly, an HA–polynucleotide complex with higher viscosity and elasticity than HA alone increased fibroblast migration and collagen synthesis [33]. However, such products differ in composition, crosslinking, particle architecture, and additives in addition to rheological behavior; therefore, these studies cannot establish a direct rheology–response relationship, and the present study does not establish equivalence or superiority relative to other filler classes.

Against this background, the main novelty of our study is not simply that a collagen filler can increase collagen production. Rather, the novelty lies in the integrated analysis of (i) ITGα5β1–ERK–cyclin D1-associated proliferation, (ii) ITGβ1–FAK–YAP-associated matrix synthesis, and (iii) ITGα11β1/Tensin-1-associated fibrillar adhesion within a single AtCOL system. Comparing 3% and 6% formulations from the same platform, together with complementary ITGβ1 loss- and gain-of-function experiments, links concentration-dependent material differences to three coordinated stages of fibroblast-driven collagen remodeling. This within-material design reduces, although does not eliminate, the compositional confounding inherent in comparisons among unrelated filler products.

Injection plane is clinically important, but the present study evaluated only subcutaneous administration. Dermal, subdermal, and supraperiosteal placement were not compared, and the model does not establish how filler–fibroblast proximity or mechanical confinement differs among planes. Therefore, the data does not support recommendations regarding deep versus superficial injection.

Within this context, potential advantages of deeper placement—including projection, structural support, persistence, or reduced superficial irregularity—remain outside the scope of the present experiments. These effects should not be inferred from the current collagen-remodeling data.

Taken together, our results suggest a limited but clinically relevant principle: material composition alone may be insufficient when formulating or selecting a collagen filler for an intended collagen-remodeling response, and oscillatory rheological properties may be one contributing factor. Prospective studies that independently control rheological and compositional variables are required before specific material properties can be recommended to maximize collagen production or guide injection-plane selection.

Several limitations should be acknowledged. Because our injections were performed only in aged mice and no young non-aged reference group was included, restoration toward a youthful state cannot be established; the results should therefore be interpreted as collagen remodeling rather than rejuvenation. Additionally, changing AtCOL concentration altered not only oscillatory rheology but also collagen content, ligand density, microstructure, degradation, and persistence, and rheology was measured at 25 ℃ without stress-relaxation, creep/recovery, or strain-amplitude testing to define the linear viscoelastic region. Furthermore, although an aged saline-injected comparator was included at 8 weeks, there was no needle-only sham group or time-matched saline group at 2 and 4 weeks, so needle trauma, early wound healing, and biomaterial-associated foreign-body responses cannot be fully separated from the AtCOL-associated changes; inflammatory and foreign-body giant-cell markers were not assessed. The commercial HDF donor age was unavailable, H_2_O_2_-induced senescence does not fully reproduce fibroblasts from aged human donors, and validation by SA-β-gal, p16, and p21 does not eliminate this limitation. Finally, the in vitro experiments used the 6% condition only; ITGβ1 overexpression efficiency requires explicit validation, the gain-of-function experiment did not include the complete FAK-YAP or ITGα11β1/Tensin-1 panel, and FAK, YAP, and α11β1 were not directly perturbed. Future work should include comprehensive time-dependent rheology, young and time-matched sham controls, aged-donor primary fibroblasts, inflammatory profiling, verified bidirectional ITGβ1 perturbation, and pathway-specific inhibition of FAK, YAP, and α11β1.

Overall, within these boundaries, the data support an association between the higher-concentration AtCOL formulation, its higher oscillatory moduli and complex viscosity, and stronger ITGβ1-centered collagen remodeling in aged mouse skin, with the principal contribution being an integrated framework that links fibroblast proliferation, matrix synthesis, and collagen assembly within one collagen system and is strengthened by complementary loss- and gain-of-function evidence.

## 5. Conclusions

The present study shows that 3% and 6% AtCOL differ in oscillatory rheological profile and are associated with distinct fibroblast and collagen-remodeling responses in aged mouse skin. Under the tested conditions, 6% AtCOL exhibited higher G′, G″, and complex viscosity and was associated with stronger ITGα5β1–ERK–cyclin D1, ITGβ1–FAK–YAP, and ITGα11β1/Tensin-1-associated responses. ITGβ1 knockdown attenuated these effects, whereas ITGβ1 overexpression produced directionally concordant downstream changes in ITGA11, fibroblast proliferation, collagen transcripts, and MMP expression. These findings support an integrated, concentration-dependent mechanotransduction model but do not establish causality for a single rheological parameter or demonstrate rejuvenation relative to young skin.

## Data Availability

The original contributions presented in this study are included in the article/Supplementary Material. Further inquiries can be directed to the corresponding authors.

## Supplementary Materials

The following supporting information can be downloaded at https://www.mdpi.com/article/10.3390/jfb17090474/s1, Figure S1: Validation of the aging phenotype by senescence-associated gene expression in skin tissues; Figure S2: AtCOL modulates MMP expression in aged skin; Figure S3: Confirmation of cellular senescence in H_2_O_2_-treated HDFs; Figure S4: ITGβ1 overexpression enhances AtCOL-associated proliferative and collagen-remodeling responses in H_2_O_2_-trated HDFs; Table S1: List of primers for quantitative real-time PCR; Table S2: List of antibodies for ELISA, WB, and ICC/IF/IHC.
