# Supplementary material for: Concentration-Dependent Rheological Properties of Atelocollagen Are Associated with Fibroblast Mechanotransduction and Collagen Remodeling in Aged Skin
Source: J Funct Biomater. 2026 Sep 17;17(9):474. doi: 10.3390/jfb17090474 (PMC13608859; doi:10.3390/jfb17090474)
Supplement: Supplementary file 1 [file jfb-17-00474-s001.zip › jfb-4472061-supplementary.pdf]

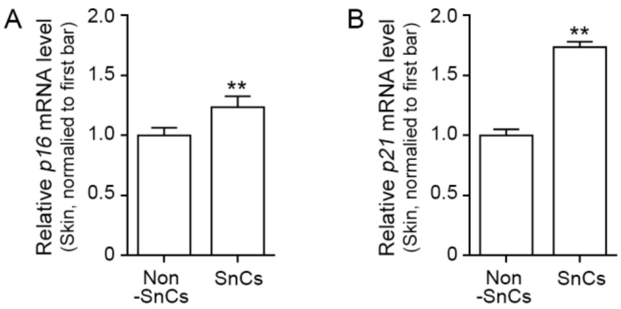

Figure S1. Validation of the aging phenotype by senescence-associated gene expression in skin tissues. (**A,B**) Relative mRNA expression levels of p16 (A) and p21 (B) in skin tissues from young and aged mice. Expression levels were normalized to actb and expressed as fold change relative to the young group (first bar). Values are expressed as mean ± standard deviation (n = 5 per group). Statistical significance was assessed using the Kruskal–Wallis test followed by Mann–Whitney U test with Bonferroni correction. ** indicates a significant difference compared with the Young group ($p < 0.01$).

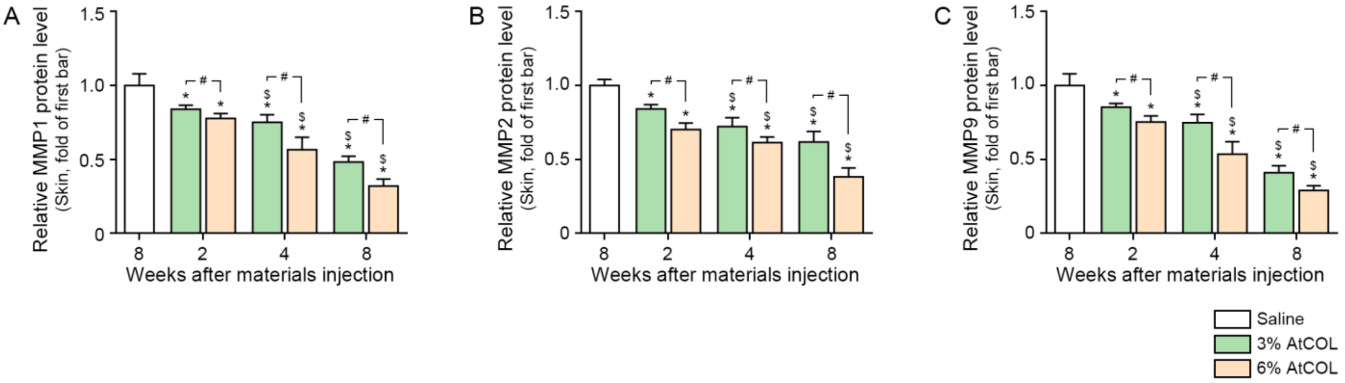

Figure S2. AtCOL modulates MMP expression in aged skin. (**A–C**) Quantitative analysis of MMP1 (A), MMP2 (B), and MMP9 (C) protein expression patterns corresponding to the western blot results shown in Figure 3F. Protein levels were normalized to β-actin and expressed as fold changes relative to the saline-treated group. Data are presented as mean ± standard deviation (n = 5). Group differences were assessed using the Kruskal–Wallis test with post hoc Mann–Whitney U tests adjusted by the Bonferroni method. *, $p < 0.05$ versus saline; #, $p < 0.05$ for 3% versus 6% AtCOL at the matched time point; $, $p < 0.05$ versus the preceding time point within the same AtCOL concentration. AtCOL, atelocollagen; MMP, matrix metalloproteinases.

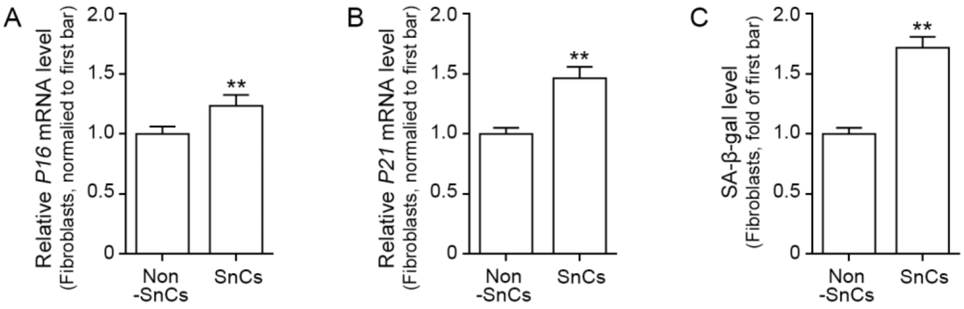

Figure S3. Confirmation of cellular senescence in H_2O_2-treated HDFs. (**A,B**) Relative mRNA expression levels of P16 (A) and P21 (B) in Non-SnCs and H_2O_2-induced SnCs. (**C**) Relative SA-β-gal signal in Non-SnCs and SnCs. Data are expressed as mean ± standard deviation (n = 5). Statistical significance was assessed using the Kruskal–Wallis test followed by Mann–Whitney U test with Bonferroni correction. ** indicates a significant difference compared with the Non-SnCs group ($p < 0.01$). HDFs, human dermal fibroblast; SnCs, senescent cells.

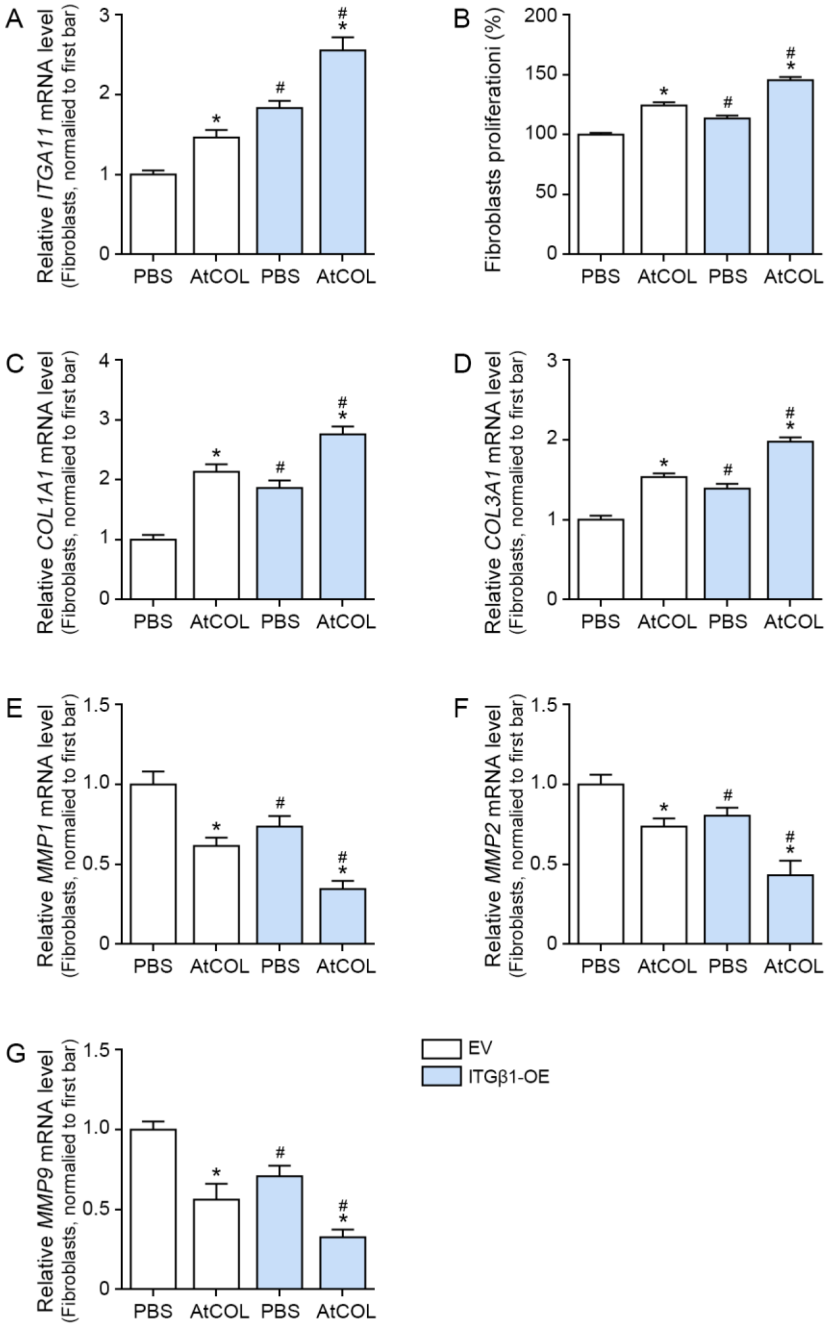

Figure S4. ITGβ1 overexpression enhances AtCOL-associated proliferative and collagen-remodeling responses in H_2O_2-trated HDFs. (**A**) ITGA11 mRNA expression, (**B**) fibroblast proliferation, (**C**,**D**) COL1A1 and COL3A1 mRNA expression, and (**E–G**) MMP1 (E), MMP2 (F), and MMP9 (G)mRNA expression in EV and ITGβ1-OE cells treated with PBS or AtCOL. For qPCR analysis, target gene expression was normalized to ACTB and expressed relative to the first bar in each panel. Data are presented as mean ± standard deviation (n = 5). Group differences were evaluated using the Kruskal–Wallis test followed by Bonferroni-corrected Mann–Whitney U test. *, $p < 0.05$, PBS vs. AtCOL within the same condition; #, $p < 0.05$, EV vs. ITGβ1-OE within the same treatment group. AtCOL, atelocollagen; COL1A1, collagen type 1 alpha 1 chain ; COL3A1, collagen type 3 alpha 1 chain; HDFs, human dermal fibroblasts; ITG, integrin; MMP, matrix metalloproteinases; OE, overexpression; PBS, phosphate-buffered saline; qPCR, quantitative real-time polymerase chain reaction.

Table S1. List of primers for quantitative real-time PCR.

**Gene *(Organism)***	**Primer sequences**	
ACTB (human)	Forward	5'-CTC GCC TTT GCC GAT CC-3′
	Reverse	5'-TCT CCA TGT CGT CCC AGT TG-3′
P16 (human)	Forward	5'-CAC TTT CCT GGG CAA CAA ATA-3′
	Reverse	5'-CTT GCG GTC ATC ATC GTA GTT-3′
P21 (human)	Forward	5'-GGA GAC TCT CAG GGT CGA AAA-3′
	Reverse	5'-GCT TCC TCT TGG AGA AGA TCA G-3′
actb (mouse)	Forward	5'-AAA GCC TAT TTC TGC CAG GAC-3′
	Reverse	5'-TCA TAG GTC ACG TAG CCC ACT-3′
p16 (mouse)	Forward	5'-GGT TCT TGG TCA CTG TGA GGA T-3′
	Reverse	5'-GCA CGA ACT TCA CCA AGA AAA-3′
p21 (mouse)	Forward	5'-ACC AGA GGC AGT AAC CAT GC-3′
	Reverse	5'-TGC TTC TAC AAA CCC ACA AAT G-3′

Table S2. List of antibodies for ELISA, WB, and ICC/IF/IHC.

Antibody	Company	Dilution rate		
		ELISA	WB	ICC/IF/IHC
β-actin	Cell signaling	-	1:1,000	-
COL1A1	Santa cruz biotechnology	1:500	-	1:50
COL3A1	Santa cruz biotechnology	1:500	-	1:50
CyclinD1	BioLegend	-	1:500	-
ERK1/2	Cell signaling	-	1:1,000	-
FAK	Cell signaling	-	1:1,000	-
ITGα5	Santa cruz biotechnology	1:20	1:200	-
ITGα5β1	Bioss	1:500	-	-
ITGα11β1	Santa cruz biotechnology	1:25	-	1:50
MMP1	FINETEST	-	1:500	-
MMP2	GeneTex	-	1:1,000	-
MMP9	ABclonal	-	1:500	-
PCNA	Abcam	-	-	1:900
pERK1/2	Cell signaling	-	1:1,000	-
pFAK	Cell signaling	-	1:1,000	-
Tensin-1	Invitrogen, Thermo Fisher	-	-	1:500
YAP	Santa cruz biotechnology	-	-	1:50

ELISA, enzyme-linked immunosorbent assay; WB, western blot; ICC, immunocytochemistry; IF, immunofluorescence; IHC, immunohistochemistry.
